# Epidemiology of acute kidney injury in children: a report from the 26th Acute Disease Quality Initiative (ADQI) consensus conference

**DOI:** 10.1007/s00467-023-06164-w

**Published:** 2023-10-24

**Authors:** Scott M. Sutherland, Rashid Alobaidi, Stephen M. Gorga, Arpana Iyengar, Catherine Morgan, Emma Heydari, A. Ayse Akcan Arikan, Raj K. Basu, Stuart L. Goldstein, Michael Zappitelli, the ADQI 26 Workgroup, David J. Askenazi, Sean M. Bagshaw, Matthew Barhight, Erin Barreto, Benan Bayrakci, O. N. Ray Bignall, Erica Bjornstad, Patrick Brophy, Jennifer Charlton, Rahul Chanchlani, Andrea L. Conroy, Akash Deep, Prasad Devarajan, Kristin Dolan, Dana Y. Fuhrman, Katja M. Gist, Jason H. Greenberg, Denise Hasson, Jennifer Jetton, Catherine Krawczeski, Leslie Meigs, Shina Menon, Jolyn Morgan, Theresa Mottes, Tara Neumayr, Zaccaria Ricci, David T. Selewski, Danielle Soranno, Natalja Stanski, Michelle Starr, Jordan Symons, Marcelo Tavares, Molly Vega, Claudio Ronco, Ravindra L. Mehta, John Kellum, Marlies Ostermann

**Affiliations:** 1 Department of Pediatrics, Division of Nephrology, Center for Academic Medicine, Stanford University School of Medicine, Palo Alto, CA USA; 2 Department of Pediatrics, University of Alberta, Edmonton, AB Canada; 3 Department of Pediatrics, University of Michigan Medical School, Ann Arbor, MI USA; 4 Department of Paediatric Nephrology, St. John’s National Academy of Health Sciences, Bangalore, India; 5 Department of Pediatrics, Section of Critical Care Medicine, Baylor College of Medicine, Texas Children’s Hospital, Houston, TX USA; 6 Department of Pediatrics, Northwestern University, Chicago, IL USA; 7 Department of Pediatrics, University of Cincinnati College of Medicine, Cincinnati, OH USA; 8 Department of Pediatrics, Hospital for Sick Children, University of Toronto, Toronto, ON Canada

**Keywords:** Acute kidney injury, ADQI, AKI epidemiology

## Abstract

The nephrology and critical care communities have seen an increase in studies exploring acute kidney injury (AKI) epidemiology in children. As a result, we now know that AKI is highly prevalent in critically ill neonates, children, and young adults. Furthermore, children who develop AKI experience greater morbidity and higher mortality. Yet knowledge gaps still exist that suggest a more comprehensive understanding of AKI will form the foundation for future efforts designed to improve outcomes. In particular, the areas of community acquired AKI, AKI in non-critically ill children, and cohorts from low-middle income countries have not been well studied. Longer-term functional outcomes and patient-centric metrics including social determinants of health, quality of life, and healthcare utilization should be the foci of the next phase of scholarship. Current definitions identify AKI-based upon evidence of dysfunction which serves as a proxy for injury; biomarkers capable of identifying injury as it occurs are likely to more accurately define populations with AKI. Despite the strength of the association, the causal and mechanistic relationships between AKI and poorer outcomes remain inadequately examined. A more robust understanding of the relationship represents a potential to identify therapeutic targets. Once established, a more comprehensive understanding of AKI epidemiology in children will allow investigation of preventive, therapeutic, and quality improvement interventions more effectively.

## Introduction

The first Acute Disease Quality Initiative focused on children (The Pediatric ADQI; pADQI) meeting was conducted in Napa, CA, USA. This inaugural pediatric meeting was the 26^th^ meeting of the Acute Disease Quality Initiative (ADQI) group (ADQI XXVI) [1]. The current manuscript entails the work performed and conclusions drawn by the Epidemiology Workgroup, one of the six a priori defined pADQI subgroups. Our focus was to explore the available epidemiologic data regarding pediatric AKI, acknowledging that, in the last 15 years, there has been significant progress in understanding the diagnosis of, risk factors for, and outcomes following pediatric AKI.

**Fig. 1:**
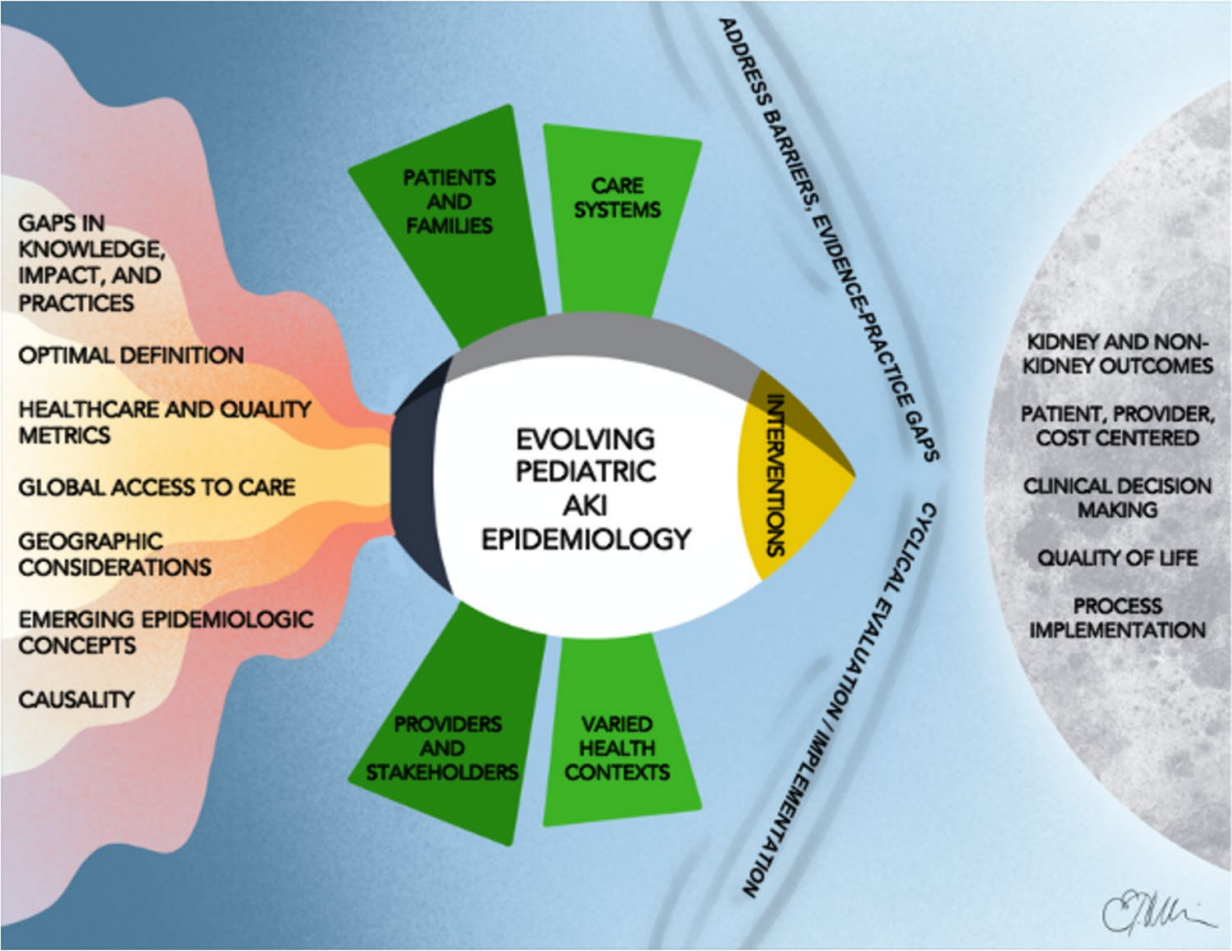
Leveraging epidemiology to improve care and outcomes in children with AKI. The application of a standard definition has led to our current understanding of pediatric AKI epidemiology. In the future, a broader, more comprehensive approach is required to bridge existing gaps. To begin, the causal relationship between AKI and outcomes remains poorly understood and needs to be explored more fully. AKI is a heterogeneous syndrome and phenotypes based on temporal and causative aspects of the disease need to be examined. Certain populations and cohorts have been well described; however, AKI in under-resourced, non-critical care, and ambulatory/community contexts remain inadequately studied; long-term functional outcomes, the impact of AKI on quality of life and healthcare utilization/costs are emerging outcomes which need investigation. AKI biomarkers may be an important component of this transition as they allow identification of AKI at the point of injury, rather than when dysfunction becomes manifest. This process will benefit from the involvement of providers and medical stakeholders as well as patients and their families; AKI should also be considered in the context of healthcare systems and resources. Ideally, this will generate an iterative process leading to improved quality and efficacy of care in addition to superior outcomes

Understanding AKI epidemiology is the foundation required to set research priorities, develop practice recommendations and implementation strategies designed to improve outcomes, and change how we care for children with AKI. A framework for how enhanced understanding of AKI epidemiology can be used to improve global AKI outcomes in an iterative manner is depicted in Fig. 1. In keeping with explicit goals of the pADQI steering committee, the seven core members of the Epidemiology workgroup deliberately included senior and junior faculty, three women and four men, individuals from both North American and non-North American facilities, and representatives from both nephrology and critical care. We herein describe our work to develop and answer the five key questions described below (Table 1).

**Table 1:** Workgroup questions and statements

Question	Statements
*Question 1: What are the knowledge gaps in our current understanding of pediatric AKI epidemiology?*	Statement 1: Pediatric AKI epidemiology is well-described in critically ill neonates and children from high-income countries; however, further study is needed in other healthcare contexts, such as low-middle income countries and in non-ICU and ambulatory settings Statement 2: Pediatric AKI studies consistently demonstrate strong associations with adverse short-term outcomes; however, the socioeconomic impact of AKI and long-term outcomes remain poorly understood
*Question 2: What are the characteristics of comprehensively described pediatric AKI epidemiology?*	Statement 3: In addition to standard characteristics, a comprehensive description of pediatric AKI epidemiology reflects current practice and best available information. As such, this information should be up to date and readily available to clinicians and institutions in real time to facilitate care of patients and families, public health surveillance, field investigations, clinical and pre-clinical studies, and policy development
*Question 3: What is needed to optimize the definition of AKI to enhance our understanding of pediatric AKI epidemiology?*	Statement 4: Optimal consensus diagnostic criteria for pediatric AKI will be based upon biomarkers of injury, rather than solely on functional assessments. Additionally, the concept of an AKI event may be expanded to incorporate diverse AKI phenotypes based upon severity and the etiologic, functional, and temporal aspects of the disease
*Question 4: In what ways can advanced epidemiologic understanding of pediatric AKI be used to catalyze scholarship and improve care and outcomes in at-risk and/or affected children?*	Statement 5: An understanding of pediatric AKI epidemiology allows us to begin employing strategies to improve primary, secondary, and tertiary AKI prevention in at-risk patient groups Statement 6: Knowledge of pediatric AKI epidemiology can catalyze the evaluation of AKI healthcare process implementation and evidence-practice gaps in order to identify relevant stakeholders, understand practice patterns, and address barriers to change in AKI care
*Question 5: In what ways does our current epidemiologic understanding of AKI support a causal relationship between pediatric AKI events and outcomes and what more is needed?*	Statement 7: Available data provide a causal link between pediatric AKI and a limited scope of short-term outcomes, supporting prevention and mitigation of pediatric AKI and its complications as valid targets for improving outcomes Statement 8: Epidemiologic understanding of pediatric AKI must evolve to generate insight that reflects the complex, nonlinear trajectory connecting AKI events in childhood with long-term health outcomes

## Methods

The pADQI consensus meeting followed the established ADQI process, as previously described, including scientific review of the literature, iterative workgroup discussions, and whole-pADQI workgroup meeting and discussions, with consensus achievement [2]. The Epidemiology workgroup was guided by a Chair and a Co-Chair, who organized video-teleconference meetings approximately quarterly, across a period of 18 months. The pADQI chairs proposed key questions, which were refined during an initial orientation and planning teleconference. Five separate video-teleconferences were held to address each of the five original key questions. Each question was assigned to two leads, who each presented a detailed summary of the current pertinent literature, identified key knowledge gaps, and formulated key statements and rationale ensuing from the workgroup discussions. After each teleconference, the Chair distributed minutes of the meeting to ensure that workgroup members could read and comment on them. At the November meeting in Napa, which included the whole pADQI group, the Epidemiology workgroup statements were presented individually and iteratively for peer review. Further refinement of the questions and statements ensued with iterative modifications continued at two subsequent web-based pADQI meetings, leading to the final Epidemiology workgroup key questions and statements below. Institutional approval was not required, and all pADQI members consented to inclusion of their work.

## Workgroup statements and rationales

The workgroup considered the questions most relevant to the refinement and evolution of our understanding of AKI epidemiology in children. Consensus statements were then developed by the workgroup and refined by the entire consensus panel. The questions posed and statements are outlined below along with the rationales underpinning the workgroup statements.

### Question 1: “What are the knowledge gaps in our current understanding of pediatric AKI epidemiology?”

#### Statement 1

Pediatric AKI epidemiology is well-described in critically ill neonates and children from high-income countries; however, further study is needed in other healthcare contexts, such as low-middle income countries and in non-ICU and ambulatory settings.

The currently accepted definition standard for AKI is the KDIGO definition [3]. The implementation of standardized criteria (i.e., KDIGO definition) to define the presence and severity of AKI has facilitated a description of pediatric AKI epidemiology. Prior to the development of a consensus definition, many different criteria were applied, leading to an epidemiological heterogeneity which created challenges for comparisons and extrapolations across different studies and populations. However, using the standardized criteria, recent prospective multinational studies provided critical insights regarding AKI epidemiology in critically ill neonates and children [4, 5]. Additionally, several observational studies described AKI epidemiology in specific cohorts of children including sepsis, those receiving nephrotoxic medications, and cardiac surgical populations [6–10]. Thus, we have a solid understanding of AKI risk factors, incidence, and outcomes in these contexts.

Despite these advances, residual knowledge gaps remain. For example, few studies have explored AKI in children receiving acute and emergency care, and minimal data exist regarding AKI in the ambulatory setting [11–17]. Additionally, we lack data regarding AKI epidemiology outside of high-income countries, although novel testing approaches and renewed interest in the applicability of the urine output AKI criteria may help address this gap [18–25]. Of note, the scarce data available describing community acquired AKI are mostly derived from populations in low-middle income countries; leveraging this advantage may help to more comprehensively describe the epidemiology of AKI in children [13, 16, 20, 26, 27].

#### Statement 2

Pediatric AKI studies consistently demonstrate strong associations with adverse short-term outcomes; however, the socioeconomic impact and long-term outcomes of AKI remain poorly understood.

Current literature describes consistent and strong associations between AKI and increased morbidity, mortality, and health resource utilization in critically ill children. Critically ill children who experience AKI are more likely to die during the period of observation, require longer duration of mechanical ventilation and hospital stay, and incur greater costs while hospitalized, and AKI survivors are more likely to receive ambulatory care and require hospitalization after discharge [4, 5, 28–30]. Importantly, the associations exhibit a gradient-response relationship between AKI severity and risk of poorer outcomes [24, 31]. More recent research has focused on assessing the medium- and long-term sequalae of AKI, including its effect on longitudinal kidney function and socioeconomic impact. Observational studies demonstrate that pediatric AKI survivors have a high prevalence of hypertension, chronic kidney disease, and proteinuria [8, 32–35]. However, prospective studies with matched controls in specific populations have not confirmed these findings consistently [36, 37]. Thus, medium- and long-term AKI survivor epidemiology requires further study to identify risk factors and potential interventions to prevent or mitigate long-term morbidity.

### Question 2: “What are the characteristics of comprehensively described pediatric AKI epidemiology?”

#### Statement 3

In addition to standard characteristics, a comprehensive description of pediatric AKI epidemiology reflects current practice and best available information. As such, this information should be up to date and readily available to clinicians and institutions in real time to facilitate care of patients and families, public health surveillance, field investigations, clinical and pre-clinical studies, and policy development.

Traditionally, AKI epidemiologic characteristics are categorized as pre-event (e.g., risk factors, key drivers, predictors), peri-event (e.g., demographics, etiology), and post-event (e.g., outcomes, recovery). However, as a clinical syndrome, many aspects of AKI remain inadequately studied. For example, studies have begun to explore epidemiologic differences between AKI that develops early vs*.* late in hospitalization as well as transient vs*.* persistent AKI [38]. The proposed definitions for these distinguishing AKI characteristics (or sub-categories or phenotypes) are still evolving. For example, the concept of persistent AKI has been defined as AKI duration above a certain number of days or even as an AKI event which is not resolved before hospital discharge [39–41]. The recent proposed addition to the AKI definition of acute kidney disease (or AKD; AKI persisting for greater than 7 to less than 90 days) has provided a new category for prolonged kidney injury, but also further increased the complexity of determining how to study AKI as a risk factor for poor outcomes. These and other new developed AKI classification refinements will need to be evaluated for associations with outcomes and in different patient populations and healthcare contexts.

AKI epidemiology is understood most comprehensively in critically ill children from high-resource settings. Future work must study other populations and locations (both in and out of hospital). Few studies have examined AKI in children receiving non-critical care, and community acquired AKI (CA-AKI) is even less well understood [12, 13, 15, 23]. While some AKI studies in low-middle income countries have been performed recently, a substantial gap remains in our understanding of kidney disease in these children [13, 16, 17, 23, 26, 27, 42]. A more robust description of AKI epidemiology in varied healthcare contexts will inform a more relevant and personalized care plan for children with AKI. Another important gap is investigation of more holistic long-term outcomes including health-related quality of life, functional status, new disability and morbidity, healthcare utilization, and financial toxicity [7, 43, 44]. Finally, in the clinical care context, current knowledge of AKI epidemiology and outcomes should be disseminated to local stakeholders to maximize the likelihood that AKI is optimally treated and followed. When AKI occurs, the ability to detect it and ascertain its presence should be optimized in order for relevant changes in management and risk mitigation to occur in a timely manner and impact patient health. Pediatric AKI epidemiological data should thus inform current practice with the best data available to the clinician in real time to treat and counsel patients and families at the bedside, enhance public health surveillance, augment field investigations and studies, and further policy development to promote child health.​

### Question 3: “What is needed to optimize the definition of AKI to enhance our understanding of pediatric AKI epidemiology?”

#### Statement 4

Optimal consensus diagnostic criteria for pediatric AKI will be based upon biomarkers of injury, rather than solely on functional assessments. Additionally, the concept of an AKI event may be expanded to incorporate diverse AKI phenotypes based upon severity and the etiologic, functional, and temporal aspects of the disease.

The current KDIGO definition relies on surrogate markers of kidney function (serum creatinine and urine output) rather than indicators of injury per se. The relationship between serum creatinine and glomerular filtration rate is not linear; more than 50% decline in functional capacity may occur before an increase in creatinine. Changes in serum creatinine after kidney injury can be delayed up to 24–48 h. There are also practical limitations to the KDIGO definition. For example, baseline serum creatinine is not always available and may require estimation or imputation. Urine output monitoring enhances AKI detection, yet application of urine output criteria is not always feasible, especially outside of intensive care settings. While the adoption of the KDIGO criteria has dramatically improved our understanding of AKI epidemiology in children, recent data underscore the benefit of kidney injury biomarkers. Molecules such as neutrophil gelatinase-associated lipocalin (NGAL), tissue inhibitor of metalloproteinase 2 (TIMP-2), and insulin-like growth factor binding protein 7 (IGFBP7) have been shown to increase rapidly in the hours following kidney injury [45]. Incorporation of the biomarkers into the definition of AKI, when used in conjunction with functional assays, will likely describe the breadth of AKI in children more precisely. As a corollary, the KDIGO definition contains urine output criteria, yet many studies do not apply them due to the aforementioned challenges. A recent review of 174 AKI studies found only 28% used urine output criteria to identify AKI [46]. However, studies show that 20% of children with AKI only meet urine output criteria and that these patients have outcomes similar to those meeting the creatinine criteria. Early studies of “sub-clinical” AKI, defined by elevated kidney injury biomarker levels but normal creatinine and urine output, have found similar associations with worse outcomes similar to those with KDIGO defined AKI [47]. Widespread adoption of injury biomarkers may help us more comprehensively describe AKI epidemiology in children [48]. The adoption of injury biomarkers within the AKI definition and further within clinical care is a much wider topic than was discussed within this workgroup’s activities. However, it was acknowledged that such progress has been somewhat slow. Reasons for this relatively slow advancement are multifactorial, including research-related and non-research-related (e.g., competing interests between different biomarkers; approval for use; commercial availability; cost) issues. From a research perspective, identifying key biomarker concentration thresholds which optimally predict outcomes when incorporated within the AKI definition and identifying which patients are most likely to benefit from having injury biomarkers measured represent key areas for investigation.

The KDIGO definition classifies AKI by stages according to the *magnitude* of change in serum creatinine and/or urine output, but new evidence suggests the *duration* of change also influences outcomes. Persistent and transient AKI may each have clinical characteristics that are associated with different outcomes [49, 50]. AKI duration and non-recovery have important prognostic implications and could provide added value in refining the definition of AKI. Finally, AKI is a complex disorder with diverse pathophysiological mechanisms based upon etiology and the molecular pathways affected. Different AKI phenotypes exist and may require different tailored approaches to treatment [51]. The KDIGO AKI definition does not incorporate this information and instead defines all AKI as a single homogeneous disease. Thus, just as the definition of AKI has been standardized, it will be important to apply consensus definitions that can be tested regarding AKI terminology (i.e., transient, persistent, community acquired) and progression (i.e., kidney recovery, acute kidney disease (AKD), and CKD).

### Question 4: “In what ways can advanced epidemiologic understanding of pediatric AKI be used to catalyze scholarship and improve care and outcomes in at-risk and/or affected children?”

#### Statement 5

An understanding of pediatric AKI epidemiology allows us to begin employing strategies to improve primary, secondary, and tertiary AKI prevention in at-risk patient groups.

A systematic and comprehensive understanding of pediatric AKI epidemiology, including incidence and risk factors for AKI and its outcomes, provides the foundation for catalyzing scholarship and the potential to lead to improved pediatric AKI outcomes. This knowledge will ultimately allow interventional studies to be designed and performed appropriately, help advocate for and educate stakeholders and patients about quality improvement opportunities, and lead to implementation of practice changes across the globe. Our current epidemiologic understanding of pediatric AKI enables deployment of strategies to prevent AKI in established, high-risk patient groups (e.g., critical illness, cardiac surgery, high nephrotoxin burden). These cohorts are at higher risk for developing AKI and should be targeted for interventional strategies designed to prevent AKI development. In many cases, prevention of the injurious event itself is not feasible. In such situations, patients at high risk for AKI or undergoing interventions associated with high risk for kidney injury can be targeted for secondary AKI prevention, where strategies are employed before or after the event to prevent AKI or mitigate its severity. Finally, our understanding of AKI epidemiology allows clinicians to identify AKI accurately in high-risk groups, so tertiary strategies can be employed with the goal of mitigating progression, severity, persistence, and chronic sequelae. As therapies, guidelines, or nonpharmacological interventions become available, the most robust epidemiological data can be used to further refine populations for intervention and target pediatric-specific outcomes.

#### Statement 6

Knowledge of pediatric AKI epidemiology can catalyze the evaluation of AKI healthcare process implementation and evidence-practice gaps in order to identify relevant stakeholders, understand practice patterns, and address barriers to change in AKI care.

Changing care for children with AKI requires adapting existing care processes, re-evaluating entrenched beliefs, and understanding the variability of resource limitations around the world. A comprehensive understanding of pediatric AKI incidence and risk factors allows exploration of relevant AKI practice patterns and healthcare resource availability which impact AKI epidemiology and its ascertainment. This, in turn, allows identification of potentially modifiable evidence-practice gaps across geographic and healthcare contexts as well as clarifies stakeholder priorities and barriers to changing AKI care. Ultimately, generated evidence will help develop opportunities for innovative AKI care process implementation. As knowledge emerges from efforts to implement novel AKI treatments or care processes, a cyclical knowledge generating process should continuously inform our understanding of pediatric AKI epidemiology and best care practice processes.

### Question 5: “In what ways does our current epidemiologic understanding of AKI support a causal relationship between pediatric AKI events and outcomes and what more is needed?”

#### Statement 7

Available data provide a causal link between pediatric AKI and a limited scope of short-term outcomes, supporting prevention and mitigation of pediatric AKI and its complications as valid targets for improving outcomes.

#### Statement 8

Epidemiologic understanding of pediatric AKI must evolve to generate insight that reflects the complex, nonlinear trajectory connecting AKI events in childhood with long-term health outcomes.

One commonly cited framework for causal inference is the Bradford Hill criteria [52]. The strength, consistency, plausibility, temporality, and biological gradient of the observed association between pediatric AKI and short term, in-hospital outcomes support establishment of causality [4, 5, 53–56]. An independent association of clinically meaningful magnitude is observed consistently in varied cohorts and settings with a dose-response relationship [5, 9, 53, 54, 57–59]. Additionally, the relationship between AKI and worse in-hospital outcomes is observed across varying definitions and study designs as well as different geographical locations [9, 42, 60–62]. Finally, when making a causal assessment of the in-hospital outcomes following pediatric AKI, it is clear that the temporal sequence supports causation, with the biological mechanisms fitting appropriately within timeframes reported for outcomes in the epidemiological literature [63–67]. Using this established framework, available data in children support a causal link between pediatric AKI and mortality, length of stay, and ventilation time, from the viewpoint of strength of association, consistency of findings, temporality, the presence of a biologic gradient, and plausibility. Identifying AKI as a contributing cause of negative short-term outcomes *implies* that its elimination will have a positive effect on pediatric health outcomes.

Although the majority of our current epidemiological understanding of pediatric AKI relates to short-term outcomes, AKI has been implicated as a risk factor for the development of CKD [33]. There appears to be a biological gradient with an incrementally increased risk of negative long-term kidney outcomes with increased severity of AKI observed using both prospective cohort methodology as well as administrative data [32, 68]. In addition, the temporal progression from AKI exposure to outcome is persuasive for causal inference and fits with the evolving exposure-to-disease paradigm [32, 33, 68]. Nephron loss, cell cycle arrest, endothelial injury, maladaptive repair mechanisms, inflammation, mitochondrial dysfunction, and epigenetic changes have all been elucidated as possible mechanisms of an AKI-to-CKD transition [69–72]. When applying the fundamental tenets of causal assessment to the relationship between pediatric AKI and adverse long-term kidney outcomes, there does seem to be biologic plausibility; however, there is not yet enough support to definitely infer causation. Unfortunately, pediatric RCTs designed to examine long-term outcomes, with experimental manipulation of AKI events, are likely to be prohibitively expensive, unrepresentative, time-limited, and subject to significant co-intervention over time. Therefore, what is currently and urgently needed to clarify the causal nature of longer-term associations in children are high-quality observational studies (including relevant comparator cohorts and much larger study samples than previously described) as well as careful consideration of, and agreement on, what defines the most meaningful and measurable longer-term outcomes after pediatric AKI.

## Summary

Our understanding of pediatric AKI has increased dramatically over the last two decades. Standard definitions have allowed us to describe the incidence of AKI in critically ill children and other high-risk cohorts. Observational studies have identified risk factors and described the ramification of AKI events and the short-term outcomes these patients experience. Despite these advances, gaps remain; we need to better understand pediatric AKI in non-critically ill children, in the ambulatory setting, and in low-middle income countries. Our ability to close these gaps may hinge on the manner in which we identify and characterize AKI events. Biomarkers may allow us to identify kidney injury rather than relying upon dysfunction as a proxy for injury. Additionally, exploring AKI phenotypes based on causality and temporality as well as studying longer-term and patient-centric outcomes will help us further refine pediatric AKI epidemiology. Ultimately, a comprehensive understanding of the AKI syndrome will allow us to implement preventative, quality improvement, and, when available, therapeutic interventions most effectively.
